# A review of pre-hospital extracorporeal cardiopulmonary resuscitation and its potential application in the North East of England

**DOI:** 10.1186/s12245-023-00581-2

**Published:** 2024-01-08

**Authors:** Dominic Jones, Fiona M. Daglish, Benjamin M. Tanner, Fergus J. M. Wilkie

**Affiliations:** Emergency Department, Northumbria Specialist Emergency Care Hospital, Northumbria Way, Cramlington, NE23 6NZ UK

**Keywords:** Pre-hospital, ECPR, ECMO

## Abstract

Patients in the UK who suffer an out-of-hospital cardiac arrest are treated with cardiopulmonary resuscitation in the pre-hospital environment. Current survival outcomes are low in out-of-hospital cardiac arrest. Extracorporeal cardiopulmonary resuscitation is a technique which is offered to patients in specialised centres which provides better blood flow and oxygen delivery than conventional chest compressions. Shortening the interval between cardiac arrest and restoration of circulation is associated with improved outcomes in extracorporeal cardiopulmonary resuscitation. Delivering extracorporeal cardiopulmonary resuscitation in the pre-hospital environment can shorten this interval, improving outcomes in out-of-hospital cardiac arrest. This article will review recently published studies and summarise studies currently being undertaken in pre-hospital extracorporeal cardiopulmonary resuscitation. It will also discuss the potential application of a pre-hospital extracorporeal cardiopulmonary resuscitation programme in the North East of England.

## Background

Patients in the United Kingdom (UK) suffering an out-of-hospital cardiac arrest (OHCA) are currently treated with cardiopulmonary resuscitation (CPR) in the pre-hospital environment. Survival outcomes remain low in OHCA, with UK figures reporting 9.5% survival with attempted resuscitation [1]. Even with high-quality chest compressions, cardiac output achieves only a minimal proportion of physiological cardiac output. Duration of time in this low-flow state is critical to outcomes, with longer times associated with greater risk of ischaemic injury to end organs, and a lower likelihood of achieving ROSC due to secondary effect of decreased coronary perfusion pressure [2].

Extracorporeal cardiopulmonary resuscitation (ECPR) is a technique which can be used to shorten the duration of low-flow states in a patient in cardiac arrest. ECPR is a treatment used for refractory cardiac arrest, defined as cardiac arrest that requires more than 10 min of CPR or more than three defibrillation attempts [3]. It is performed by passing the patient’s blood through a membrane oxygenator and returning it under pressure into the arterial system. ECPR improves cardiac output and oxygenation, leading to decreased chances of ischaemia and hypoxia with irreversible end organ damage before ROSC is established [4, 5]. In addition, decreasing the duration of the low-flow state by reducing the time to ECPR and coronary reperfusion has been shown to improve neurological outcomes in OHCA [6].

ECPR is only useful in altering outcomes if it can be administered before irreversible ischaemia has occurred. This means that the time between onset of cardiac arrest and provision of ECPR should be as short as possible and preferably less than 60 min [7], with prompt and high-quality chest compressions given during this time [4].

Three recently published randomised controlled trials comparing in-hospital ECPR treatment with conventional CPR treatment for OHCA have shown varying results. The ARREST trial showed a significantly greater survival to hospital discharge in patients treated with ECPR. The trial was abandoned early due to the posterior probability of ECPR superiority exceeding the prespecified boundary [8]. The INCEPTION and Prague OHCA trials showed no statistically significant difference in hospital survival. However, they had median times to ECPR of 74 min and 61 min respectively [9, 10].

Currently, in the UK, there are only six centres with the capability to provide ECPR [11]. Even in areas that provide ECPR, OHCA patients usually have an extended period in a low-flow state before ECPR could be established, leading to poorer outcomes. Pre-hospital ECPR aims to shorten this time by getting trained medical practitioners and equipment to the patient in the least possible time. This paper will review the current literature on pre-hospital ECPR and how this may apply to the development of a pre-hospital ECPR service in the North East of England.

### Extracorporeal membrane oxygenation in the UK

Extracorporeal membrane oxygenation (ECMO) was first established in the UK at the Glenfield Hospital Leicester in 1989 with support from the Heart Link Children’s Charity [12]. ECMO in the UK is a national service that is commissioned and funded centrally by the National Health Service (NHS). There are five adult centres in England and one satellite centre in Scotland that provide ECMO support to their respective regions: University Hospital of South Manchester, Glenfield Hospital (Leicester), Royal Papworth Hospital (Cambridge), Royal Brompton & Harefield Hospital (London), Guys and St Thomas’s Hospital (London) and Aberdeen Royal Infirmary (Scotland) [12]. Referral of patients from Northern Ireland and Wales is managed on an ad hoc basis by English centres with the Glenfield Hospital working with its satellite partner in Aberdeen covering Scotland [11, 13, 14].

Prior to the COVID-19 pandemic, referrals were made directly to regional centres. However, in preparation for the anticipated increase in patients requiring ECMO, a centralised referral service was created, and this remains in place today [13]. Exclusion criteria set out by NHS England include patients who have been in prolonged cardiac arrest, defined as greater than 15 min, making successful referral of OHCA patients more difficult [13]. Prior to the 2019 COVID pandemic, there were 15 ECMO beds available nationally; however, this was increased to 100 during the pandemic [15]. A recent review of the service indicates that there is capacity to deal with surges such as the COVID-19 pandemic, suggesting that there may also be capacity for patients requiring ECPR [16].

### Global trends in pre-hospital extracorporeal cardiopulmonary resuscitation

Since 1990, the number of ECMO capable centres globally has been increasing, with 557 ECMO centres registered in 2022 [17]. In 2022, these centres registered a total of 14,509 adult ECPR cases, a vast increase from 2009 where there were fewer than 100 cases [17, 18]. Most of these cases involved in-hospital ECPR with pre-hospital cases being the minority. Of all adult ECPR cases, there was a reported 30% (4463) survival to transfer or discharge [19]. As of 2020, the areas with highest reported ECPR use were South Korea and Japan followed by Taiwan, North America and Europe [20]. At the time of writing, there are a small number of established pre-hospital ECPR programmes, described below.

#### Paris, France

ECPR was first established in France at the Service d’aide médicale urgente de Paris in 2011, and since then, it has expanded to several other cities. The service is based around the mobile intensive care unit (MICU), an established part of French pre-hospital care. These units can provide all equipment required as though they were in-hospital to either ‘see and treat’ on scene or ‘scoop and run’ to take the patient to hospital. The mobile ECPR team consists of an anaesthetist-intensivist or emergency physician, an anaesthetic nurse and a paramedic. This team is available 24 h a day, 7 days a week [21]. The ECPR team is dispatched to all witnessed cardiac arrests alongside the MICU and a basic life support unit. The ECPR team is then stood down if there is ROSC or no indication for ECPR. The objective of the ECPR team is to establish patients on an ECMO circuit within 60 min of the witnessed arrest and convey them to hospital for further care [21].

#### Albuquerque, USA

The pre-hospital ECPR service in Albuquerque was setup in 2019 by the University of New Mexico Hospital in collaboration with the Albuquerque fire and rescue service. The pre-hospital ECPR team consists of a critical care cannulating physician and two fire and rescue paramedics trained in assisting cannulation that work from a specialised ambulance equipped to allow on-scene ECMO to take place. Dispatch relies on patients having a witnessed cardiac arrest, being identified by attending paramedics as a suitable candidate for ECMO and being greater than 35 min away from hospital. The time limit was decided as those less than 35 min away could be established on a circuit in-hospital within 60 min of collapse [22].

#### Netherlands

At present, the On-Scene trial in the Netherlands is equipping all the helicopter emergency medical services (HEMS) with ECPR capabilities. When a witnessed cardiac arrest occurs, two ambulance crews, police and fire crews are called to initiate superior quality CPR. HEMS crew is also dispatched; if the patient meets the criteria for ECPR, the scene is protected by the police and fire crew, whilst the HEMS team establish the patient on ECMO. Once established on the circuit, the patient is transported by land ambulance to the nearest ECMO capable hospital [23].

#### Regensburg, Germany

In Regensburg, the ECPR team and the ambulance service are alerted simultaneously to patients suffering OHCA. Conventional CPR is initiated then; following a briefing, a decision to initiate ECPR is made by the ECPR team. Indications for ECPR include a witnessed cardiac arrest, bystander CPR and less than 60 min of CPR prior to initiation of ECPR. Patients with known terminal malignancy, traumatic cardiac arrest and unwitnessed cardiac arrest are excluded [24].

### Patient selection and inclusion criteria

Current pre-hospital ECPR programmes use strict acceptance criteria. The Sub30 trial is an ongoing UK-based trial in pre-hospital ECPR. Their inclusion and exclusion criteria are detailed in Table 1 [25]. These are the only pre-hospital ECPR criteria currently suggested for the UK population. In 2021, the Extracorporeal Life Support Organization (ELSO) published a consensus statement with suggested inclusion criteria for ECPR shown in Table 2 [18].

**Table 1:** Inclusion and exclusion criteria for Sub30 study [25]

Inclusion criteria	Exclusion criteria
Presumed cardiac aetiology of arrest	Visibly < 18 or > 65 years old
Witnessed OHCA	Known or visible advanced pregnancy (when resuscitative hysterectomy should be performed)
Received bystander chest compressions within 3 min	No signs of life (physical movement or breathing) AND Evidence of ineffective chest compressions suggested by the following: • The absence of electrical activity at 20 min time-out • End-tidal carbon dioxide level of < 1.3 kPa (10 mmHg)
Remain in cardiac arrest at 20 min following the call to emergency services OR fail to sustain ROSC in the pre-hospital setting	ECMO unlikely to benefit patient (e.g. advanced malignancy, severe frailty)

**Table 2:** Inclusion and exclusion criteria from ELSO consensus statement [18]

Inclusion criteria
Age < 70 years
Received bystander CPR within 5 min
Initial cardiac rhythm of ventricular fibrillation/ventricular tachycardia/pulseless electrical activity
Arrest to ECMO flow < 60 min
End-tidal carbon dioxide > 1.3 kPa (10 mmHg)
Intermittent ROSC or recurrent VF
Absence of life limiting comorbidities
No known aortic valve incompetence

The criteria suggested in the Sub30 trial and those by the ELSO are comparable. The main differences are the lower upper age limit and the shorter time to bystander CPR in the Sub30 trial. The authors do not provide any evidence for this, but it can be assumed these changes would aim to improve survival. The Sub30 trial does not include initial cardiac rhythm as an inclusion criteria. However, the inclusion criteria of presumed cardiac aetiology is linked to the initial cardiac rhythm, as cardiac aetiology is more likely to present with ventricular fibrillation or ventricular tachycardia. The ELSO suggested criteria of no known aortic valve incompetence would be difficult to ascertain in the majority of cases in the pre-hospital environment.

Having strict inclusion criteria limits those who can receive ECPR to those who are assumed more likely to survive. This aims to balance resource-associated cost to survival likelihood and to improve survival statistics overall. Broadening these inclusion criteria may give more patients a chance of survival but may worsen the neurologically intact survival rate. As techniques advance and complication rates decrease, reassessing ECMO suitability criteria may be appropriate.

Koen et al. [26] reviewed current ECPR protocols between 2009 and 2019, with the aim to improve standardisation and to identify the best protocol for patient survival. There is controversy regarding the impact of age on outcomes of ECPR. Survival has not directly been shown to be linked with advancing age [27]. Factors such as baseline functional status and comorbidities are important considerations that indicate prognosis when initiating ECPR, and age is often associated with these. In the pre-hospital setting, this information is often limited, and advanced age is used as a proxy to rule out patients assumed to have a poor functional baseline. However, their data suggests using age as an exclusion criterion may not be appropriate [27].

### Access techniques

ECMO can be provided by a central or peripheral route. Central access is an invasive surgical procedure requiring opening of the chest via sternotomy and insertion of catheters into the right atrium and aorta. Due to a high complication rate and extensive resource use, central access is not currently deemed viable in the pre-hospital environment [28]. Peripheral cannulation can be performed using percutaneous Seldinger technique, open cut down Seldinger (‘semi-Seldinger’) or open cut down with end to side graft to the artery [29]. Typically, the femoral artery and either the femoral vein or internal jugular vein are used as access sites [30]. Although evidence for comparison of differing cannulation methods in the pre-hospital environment is limited, current trials examining the potential provision of pre-hospital EPCR favour the Seldinger or semi-Seldinger approach in their protocols, either with or without ultrasound guidance [22, 25, 31].

### Review of recently published evidence

A MEDLINE literature search was conducted to include English language papers published since 2018 that report the implementation of pre-hospital ECPR. Further studies were identified using a structured Internet search utilising the same terms.

The search terms used were ‘prehospital and extracorporeal membrane oxygenation’, ‘prehospital and extracorporeal cardiopulmonary resuscitation’, ‘pre-hospital and extracorporeal membrane oxygenation’ and ‘pre-hospital and extracorporeal cardiopulmonary resuscitation’. This search produced a total of 224 abstracts, of which 6 publications were suitable. Table 3 displays a summary of the results of these publications.

**Table 3:** Results of MEDLINE literature search reporting the implementation of pre-hospital ECPR since 2018

Reference	Study type	No. of pre-hospital ECPR patients	Mean low-flow time	No. of patients who survived to discharge
Bougouin, et al. (2019) [32]	Prospective registry study	135	Not reported	19
Marinaro et al. (2020) [22]	Case report	1	40 min	0
Fujita et al. (2020) [31]	Case report	2	40 min	2
Petermichl et al. (2021) [24]	Retrospective case–control study	69	48 min	21
Hutin et al. (2021) [33]	Retrospective case–control study	33	110 min	5
Pozzi et al. (2022) [34]	Prospective case–control study	30	71 min	7

The above publications included 270 cases of pre-hospital ECPR. A total of 54 (20%) patients survived to discharge. The most important outcome measure reported in the studies is survival with a favourable neurological outcome. A cerebral performance category (CPC) of 1 or 2 at discharge from hospital is used to indicate this [35]. Table 4 displays the different CPCs. Unfortunately, the study by Bougoin et al. (2019) [32] did not report the neurological status of survivors. However, of the 35 survivors in the other included studies, 30 (85.7%) survived with a favourable neurological outcome.

**Table 4:** Glasgow-Pittsburgh cerebral performance categories [35]

CPC 1	Full recovery or mild disability
CPC 2	Moderate disability but independent of ADLs
CPC 3	Severe disability and dependent on others for ADLs
CPC 4	Coma or vegetative state
CPC 5	Brain death

The largest included study by Bougouin et al. (2019) [32] aimed to compare the outcomes of OHCA patients treated with ECPR and conventional CPR in Paris. A total of 13,191 patients were included in the study. A total of 525 patients were treated with ECPR, and of these, 136 patients were treated with pre-hospital ECPR. The authors found that pre-hospital ECPR was independently associated with hospital survival to discharge (odds ratio 2.9; 95% *CI* 1.5–5.9, *p* = 0.002) and survival with a good neurological outcome (odds ratio 2.9; 95% *CI* 1.3–64, *p* = 0.008). The authors suggest that this is due to the decreased low-flow time. This conclusion is in keeping with previously published data on ECPR. In addition, the authors found that initial shockable rhythm and transient ROSC during initial resuscitation were independently associated with hospital survival to discharge in patients treated with ECPR. They suggest that these two factors could form part of future inclusion criteria for ECPR. Overall, the results of this study suggest that pre-hospital ECPR is associated with better outcomes. However, the small sample size means the conclusions need to be viewed with caution. In addition, the fact that the implementation of pre-hospital ECPR is well established in Paris means these conclusions may not be applicable to areas with less established pre-hospital ECPR protocols [32].

Petermichl et al. (2021) [24] undertook a retrospective analysis of 69 cases of pre-hospital ECPR in Regensburg, Germany, assessing the reliability of prognostic biomarkers. Twenty-one (30.4%) patients survived to hospital discharge, with 17 (24.6%) surviving with a favourable neurological outcome. The authors found that the time to ECPR was significantly shorter for the CPC 1–2 groups compared to nonsurvivors (40 min vs 47 min, *p* = 0.010). There was no statistically significant difference in the time to ECPR between survivors with a favourable neurological outcome and those without. This study shows improved survival with shorter low-flow times in pre-hospital ECPR. The small sample size and single-centre setting of the study mean that their conclusions need to be viewed with caution [24].

Hutin et al. (2022) [33] conducted a retrospective analysis of a helicopter-borne ECPR team in the Ile-de-France region around Paris. The team was created to reach patients presenting with OHCA in areas with a greater than a 60-min transfer time to the nearest ECPR centre. Thirty-three patients were included; of these, 5 patients (15.1%) survived to discharge with a good neurological outcome. Unfortunately, despite the use of a helicopter, the authors were only able to achieve a mean low-flow time of 110 min. However, the survival rate of 15.1% was still higher than the 4% survival rate given by Le Guen et al. (2011) [36] for in-hospital ECPR in patients presenting with a mean low-flow time of 120 min. Again, the small sample size means further work is required to obtain reliable conclusions. Nonetheless, the use of a helicopter-borne team improved equity of access to ECPR for patients who had a cardiac arrest in more remote locations [31].

Pozzi et al. (2022) [34] published preliminary data from 30 pre-hospital ECPR cases, conducted between June 2017 and December 2021 in Lyon. A total of 7 (23.3%) survived to hospital discharge with a good neurological outcome. The authors then followed up survivors past discharge and found that at a mean follow-up time of 27.5 months (range 3.1–57.1 months), all survivors were still alive and maintained a good functional and neurological status. As with the previously discussed studies, the small sample size is the main limitation [34].

#### Reported complications

The main complication reported in the included publications was cannulation failure. A total of 19 (9.5%) instances of failed cannulation were reported [22, 31–34]. This is comparable to previously reported in-hospital failed cannulation rates of 5–10% [37–39]. It can be expected that in the pre-hospital environment, the failure rate would be higher than in-hospital. The reasons for this could range from environmental factors such as weather and lighting, lack of ultrasound equipment and the lack of a larger team of alternative trained cannulators that may be available in hospital. The only study to look at additional complications was Pozzi et al. (2022) [31]. They reported 3 (11.5%) cases of lower limb ischaemia. Again, this is comparable to previously published data on in-hospital ECMO of 12–15% [34, 37, 38]. In addition, they reported 2 (7.6%) cases of cannulation site infection requiring surgical debridement, which is comparable to previously reported rates of 10% in the in-hospital population [34, 37, 38]. However, it can be expected that the rate of cannula infections in pre-hospital ECPR would be higher than those in-hospital due to the less sterile environment. The most common complication reported during in-hospital ECMO is haemorrhage, with major bleeding events requiring surgical intervention being reported in 27% of cases [34]. In the pre-hospital setting, the morbidity and mortality associated with these major bleeding events could be significantly higher, due to the lack of access to large volumes of pre-hospital blood and immediate radiology and surgical intervention. However, there is no published data to quantify these risks.

#### Organ donation

Another significant role of ECPR is the potential to increase organ donation rate in patients where it is unsuccessful. Studies have shown that ECPR produces a larger number of suitable organ donors [30, 40, 41]. This is due to the higher prevalence of patients suffering brain death when treated with ECPR, compared to those treated with conventional CPR [33, 42]. Hutin et al. (2022) [33] found that 18% of patients treated with ECPR developed brain death and were successful organ donors [33]. Marinaro et al. (2020) [22] reported one case of brain death; however, the patient was deemed an unsuitable candidate for organ donation [22]. In addition, the reduction of ischaemic time is important for successful organ donation [30]. Although no studies have evaluated the link between pre-hospital ECPR and successful organ donation, it can be theorised that the reduction in low-flow time offered by pre-hospital ECPR would reduce ischaemic time.

### Ongoing clinical trials

The use of pre-hospital ECPR requires further research to assess its effectiveness and suitability, as currently there is limited data to reach reliable conclusions [43]. Ongoing clinical trials into the use of pre-hospital ECPR have been reviewed below.

#### Sub30

Sub30 is a trial in Greater London, UK, undertaken by Barts and the London NHS Trust. Sub30 commenced in September 2019, with an estimated completion date of December 2022. No study results are currently published [25]. The aim of the study is to assess whether ECPR can be initiated within 30 min in the Greater London region. The primary outcome is to measure the proportion of patients receiving pre-hospital ECPR within 30 min of witnessed collapse. Secondary outcome measures include factors which limit time to ECPR, complications of ECPR and survival to discharge with a favourable neurological outcome.

Estimated patient enrolment in Sub30 is six patients. Criteria for inclusion in the study are shown earlier in Table 1. The Sub30 study protocol explains that for cases where ECPR is unable to be initiated, for example in locations where the study did not cover geographically, patient outcomes without ECPR will be compared.

If the Sub30 trial shows pre-hospital ECPR can be safely set up within 30 min of witnessed patient collapse, the authors hope to conduct a larger efficacy study. Using a small patient number does reduce reliability of results; however, given this is a feasibility study, the numbers are appropriate. When considering if the findings could be applicable to the rest of the UK, it is worth noting that this study was conducted in Greater London; rural areas will have a further distance between patients and ECMO centres, which may make meeting this 30-min time cut off more difficult.

#### On-scene

The Erasmus Medical Centre in the Netherlands is sponsoring the On-Scene trial, aiming to identify if there is a difference in survival with pre-hospital HEMS providing standard CPR versus HEMS with ECPR in OHCA [44].This study has an estimated enrolment of 390 participants. It commenced in October 2021 and aims to be completed in January 2026.

Primary outcome measures include patient survival to hospital discharge, percentage of patients with favourable neurological outcome at 6 to 12 months following cardiac arrest and cost-effectiveness. Pre-hospital ECPR, hospital-initiated ECPR and no ECPR survival rates are also to be compared. If enough data is collected, this should provide reliable conclusions on whether the addition of ECPR affects survival in those who meet the selection criteria. As this study is being conducted in the Netherlands, it is worth considering if the data may be extrapolated to the UK.

#### APACAR2

APACAR2 is a Parisian study that has sought to compare survival outcomes in pre-hospital and in-hospital ECPR [45]. It was completed in 2020 with 65 patients enrolled in total. The study is yet to be published as of May 2023. The study aimed to prove a significant improvement in survival with a favourable neurological outcome by initiating pre-hospital ECPR. The authors have hypothesised that pre-hospital ECPR will result in 20% survival compared to less than 5% in those receiving in-hospital ECPR. Secondary outcomes will compare the rates of successful cannulation, complications and organ donation rates between the two groups. Again, the limited patient numbers may produce less reliable conclusions. The 65 patients enrolled are less than the expected enrolment of 210 patients based on the authors power calculations. The same points previously discussed about geographical differences between Paris and parts of the UK may limit the applicability of results to the UK.

### Developing a pre-hospital ECPR programme for the North East of England

Healthcare in the UK is largely nationalised and provided free at the point of contact for all. In the North East, there are seven acute NHS Foundation Trusts and one Ambulance NHS Trust, the North East Ambulance Service (NEAS) [46]. The region’s pre-hospital care services are supported by the air ambulance charity, the Great North Air Ambulance Service (GNAAS).

The North East of England covers an area of 8272 km^2^ [47]. Whilst most of the population in the North East live only 8.25 km from their admitting emergency hospital [48], the designated adult ECMO centre for the North East is the Glenfield Hospital (Leicester) which is anywhere from 264 to 395 miles by road depending on your location in the region [49, 50]. Even if patients were immediately taken by GNAAS helicopter and flew at their maximum speed, initiating ECPR within 60 min of OHCA would be extremely difficult [51]. Having a team locally that could initiate pre-hospital ECPR prior to transfer could improve patient outcomes.

In the year 2019, there were 1951 OHCAs that had resuscitation [52] attempts performed by NEAS; of these, only 7.7% survived to discharge from hospital against a national average of 9.5% [53]. It must be stressed that not all of these patients would have been suitable candidates for pre-hospital ECPR; however, there is a clear need for improvement in survival given it is well below the national average. The North East region is home to 9 of the 20 most vulnerable left behind areas (LBA) in the UK, which are severely economically and socially deprived [54]. LBA have consistently worse rates of cardiovascular disease when compared to the national average [54]. This increased disease burden and economic deprivation are both likely to contribute to worse cardiac arrest outcomes for the North East [55, 56].

### Planning for ECPR

The basis of success for previous pre-hospital ECPR programmes around the world has been an integrated multidisciplinary and multiagency approach. Creating a successful programme in the North East of England will require the same approach. A description of proposed agencies and bodies are required, and their roles are described below.

### Great North Air ambulance service

GNAAS covers the north of England and supports NEAS with air ambulance rotorcraft and road vehicles that are staffed by a consultant pre-hospital doctor and specialist pre-hospital paramedic. This provides both an appropriate level of expertise to carry out ECPR as well as speed of transport to allow a patient to be established on ECMO within 60 min.

An agreement from GNAAS to allow for its clinicians to be trained to carry out ECMO cannulation and establish patients on ECPR will be required. Collaboration between teams in the UK and countries such as Paris and the Netherlands with established pre-hospital ECPR programmes may be needed for training. Coordinating portable ECMO circuits on current GNAAS rotorcraft and land vehicles would also be needed. GNAAS is a charitable organisation, relying on them to fund both ECMO equipment for ECPR, and training for their staff is likely to be unsustainable for the long term. Therefore, discussions with NHS England will be required to establish where funding is to come from.

### North East Ambulance Service NHS Trust

NEAS is the dedicated ambulance service for the North East region. They deliver both the NHS 111 and 999 emergency service and provide an unscheduled care service that responds to emergency calls; this includes dispatch of both ambulances and rapid response vehicles [57]. We would propose that NEAS take a similar role to the Dutch ambulance service in the On-Scene trial; on notification of a cardiac arrest, the emergency operations centre would dispatch an ambulance crew or rapid response vehicle to the scene, alert the police and fire service to aid and alert a GNAAS ECPR team. The NEAS Ambulance crew would be able to initiate superior quality CPR reducing no flow time, whilst they await arrival of the GNAAS ECPR team. They could also begin identifying whether the patient is a candidate for ECPR, and if the patient is not appropriate, communicate with the GNAAS team to stand them down. One potential issue is over-triage of potential candidates for ECPR, leading to GNAAS being dispatched inappropriately. As there are only two rotorcraft covering the entire North East region, they need to be dispatched appropriately as this could be a significant resource burden on GNAAS. There will need to be strict criteria for NEAS dispatchers to activate the pre-hospital ECPR team.

The already established NEAS operations centre and its working relationship with the fire, police services, GNAAS and local first responders make them the ideal party to co-ordinate the mobilisation of ECPR services.

### Acute NHS trusts

Once eligible patients are established on ECPR, they must be taken to a centre providing primary PCI or an ICU with ECMO capabilities. An agreement must be made with NHS trusts in the region providing primary PCI to accept ECPR patients. Critical care units in these trusts alongside the cardiothoracic and cardiology teams must be familiar with managing patients on ECPR. There are no such links, and conversations about such a collaboration have not been formally discussed.

With the Glenfield Hospital as the main accepting centre for North East ECMO patients, there will need to be an agreement for them to accept ECPR patients following their treatment with primary PCI locally or for direct transfers from the pre-hospital ECPR team if primary PCI is not indicated.

## Conclusions

The rationale that pre-hospital ECPR can reduce low-flow time in refractory cardiac arrest and therefore improve survival with good neurological outcome in OHCA has been the main driving force behind its implementation. This rationale appears to be supported by published data; however, it is clear more research is needed to draw reliable conclusions. Data from forthcoming trials that are looking directly at the effect of ECPR on survival will provide useful evidence to aid the development of ECPR services in the UK. The results from European studies may be extrapolated to the UK, taking into consideration variations in health care systems and geographical impact.

The development of pre-hospital ECPR programmes has progressed rapidly in recent years. Currently, the NHS ECMO network has the potential capacity, expertise and geographical distribution to support patients who have been placed on ECPR in the pre-hospital environment. Additional work is clearly needed to assess the cost-effectiveness of pre-hospital ECPR in the UK. In addition, the North East of England has all the pre-requisite organisations to support an ECPR service. However, it will require close co-operation between all the above parties and significant additional funding to make this service a reality.

## Data Availability

Data sharing is not applicable to this article as no datasets were generated or analysed during the current study.

## Abbreviations

CPC: Cerebral performance category
CPR: Cardiopulmonary resuscitation
ECMO: Extracorporeal membrane oxygenation
ECPR: Extracorporeal cardiopulmonary resuscitation
ELSO: Extracorporeal Life Support Organization
GNAAS: Great North Air Ambulance Service
HEMS: Helicopter emergency medical services
LBA: Left behind area
MICU: Mobile intensive care unit
NEAS: North East Ambulance Service
NHS: National Health Service
OHCA: Out-of-hospital cardiac arrest
PCI: Percutaneous coronary intervention
ROSC: Return of spontaneous circulation
UK: United Kingdom
